# Case Report: Does the misplaced titanium mesh cage after total spondylectomy causing cervicothoracic cord compression need to be removed during revision surgery?

**DOI:** 10.3389/fsurg.2024.1394135

**Published:** 2024-10-17

**Authors:** Xin Wang, XiaoFei Cheng, Jie Zhao, ChangQing Zhao

**Affiliations:** Shanghai Key Laboratory of Orthopaedic Implants, Department of Orthopaedic Surgery, Shanghai Ninth People’s Hospital, Shanghai Jiao Tong University School of Medicine, Shanghai, China

**Keywords:** mechanical failure, cervicothoracic junction, total spondylectomy, cage subsidence, revision surgery

## Abstract

**Background:**

Mechanical failure following total spondylectomy is a surgical challenge. The cervicothoracic junction region is a special anatomical site with complex biomechanics, and few studies have reported a detailed surgical management strategy for cases where the mesh cage subsides and compresses the spinal cord in the cervicothoracic junction region after total spondylectomy.

**Case presentation:**

A 56-year-old male patient experienced screw and rod fracture and mesh cage retropulsion into the spinal canal 5 years after total spondylectomy for osteochondroma in the first to third thoracic vertebrae. The patient complained of numbness and discomfort in both lower extremities, accompanied by unstable walking for 8 months prior to admission at our hospital. We concluded that uncorrected local kyphosis in the cervicothoracic junction after the first surgery resulted in current mesh cage subsidence and rod/screw fracture. Considering the difficulty and risks of removing the mesh cage from the anterior approach, we initially freed the superior end of the mesh cage without removing the mesh from the anterior approach by resecting the C6/7 intervertebral disc and the destroyed C7 vertebral body. We then removed the original screws and rods and performed long segment fixation from C4 to T6 via a posterior approach after recovering sagittal alignment by skull traction. Finally, the iliac bone was harvested and transplanted between the superior end of the mesh cage and the inferior end plate of C6 to fill the defect caused by kyphosis correction and C7 vertebral resection. After surgery, the patient experienced sagittal alignment reconstruction and symptom relief, and he was asked to wear a cast for at least 6 months until bone fusion was achieved. At the 3-year follow-up, there was fusion between the mesh cage and the C6 vertebra with successful instrument reconstruction and no mesh cage subsidence were observed.

**Conclusions:**

When a subsided and migrated titanium mesh cage is difficult to remove after mechanical failure following total spondylectomy, recovering sagittal alignment to achieve indirect decompression based on unique anterior and middle column reconstruction, solid instrument construction, and bone fusion is an alternative solution.

## 1 Introduction

Total spondylectomy involves the complete removal of a vertebral tumour via a three-column spine resection, including the vertebral body, lamina, disc, and facet joint (1). Due to severe instability after total spondylectomy, metal instrument construction via anterior and middle column support combined with posterior long segment fixation is performed to achieve spinal reconstruction and prevent the tension-band effect and pseudarthrosis. If necessary, additional anterior internal fixation can sometimes be performed. Subsequent mechanical failure is common in patients with a long life expectancy. However, to avoid secondary revision surgery, surgeons generally attempt to perform combined anterior and posterior surgeries and long segment fixation when formulating the initial surgical strategies (2). Once mechanical failure occurs, the operability of revision surgery is limited and more difficult than that of other spinal surgeries.

The cervicothoracic junction (CTJ) is an anatomical site with complex biomechanics. Surgery in this area is challenging because of the limited working area and low fault tolerance for avoiding injury to the mediastinum (3). To the best of our knowledge, few studies have reported a surgical protocol to address mesh cage subsidence and retropulsion after total en bloc spondylectomy (TES), especially in the cervicothoracic region, despite several studies investigating the risk factors for instrumentation failure after TES (4–6). Herein, we present the case of a 61-year-old male with severe spinal cord compression caused by mesh cage subsidence and retropulsion due to wear of the C7 vertebral body and local kyphosis in the cervicothoracic region. We demonstrate a strategy to relieve compression by retaining the mesh cage when it is difficult to remove and replace.

## 2 Case description

**Figure 1:**
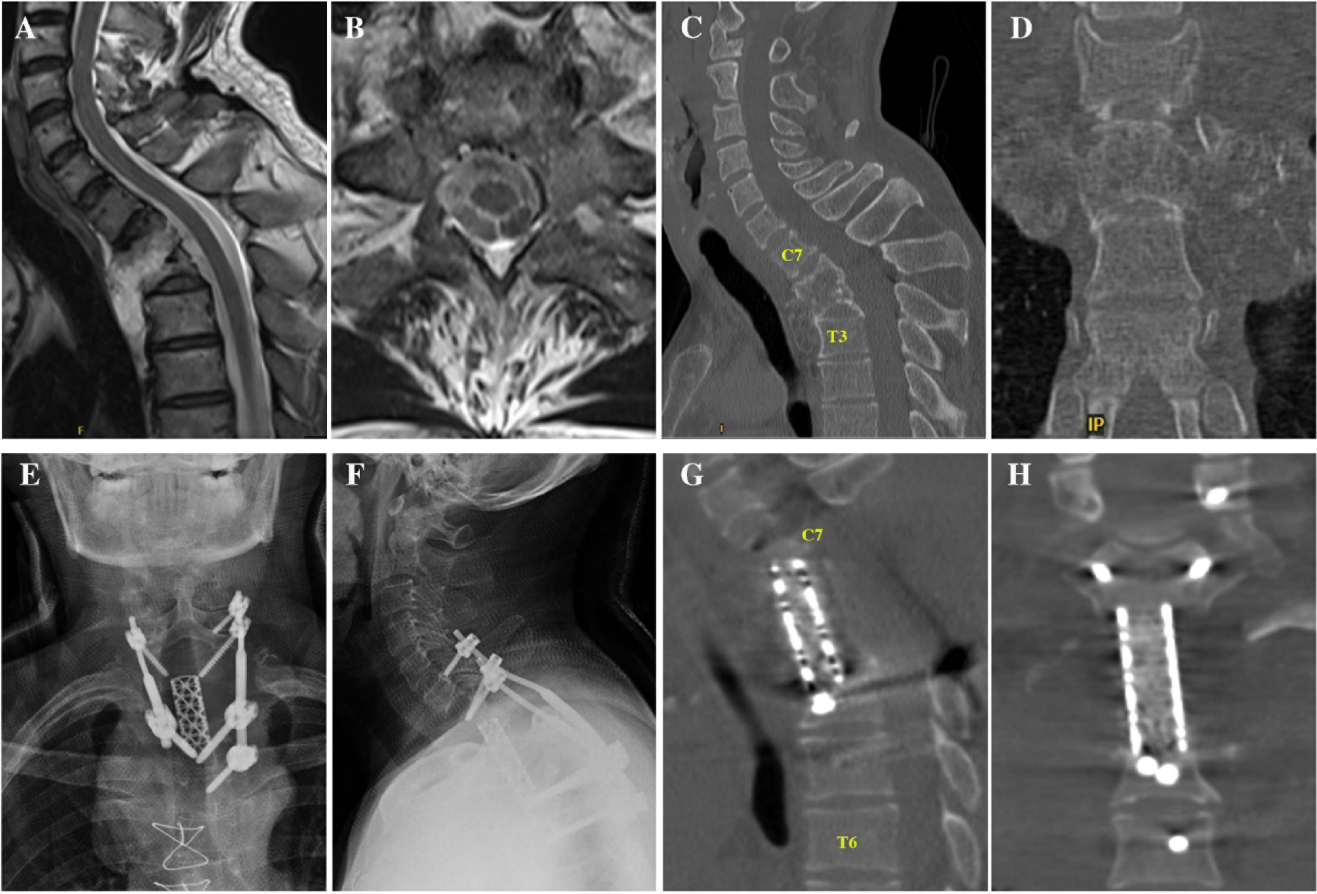
**(A,B)** Sagittal and axial MRI showing a tumour in the T1-2 kevel without obvious spinal cord compression. **(C,D)** Sagittal and axial CT showing tumour invasion of the T1–3 vertebral bodies. **(E,F)** Anteroposterior and lateral x-rays showing the region after total spondylectomy. **(G,H)** Sagittal and coronal CT image after total spondylectomy. CT, computed tomography; MRI, magnetic resonance imaging.

In 2014, a 56-year-old male with a 4-year history of no sweating on the left facial area presented to the hospital and underwent combined anteroposterior surgery for the treatment of an osteochondroma located in the first to third thoracic vertebrae (Figures 1A–D). During the first surgery, the T1–T3 vertebrae were resected and a titanium mesh cage filled with autologous iliac bone was used for anterior support. A pedicle screw/rod system at C6, C7, T4, and T5 was used for posterior support (Figures 1E–H).

**Figure 2:**
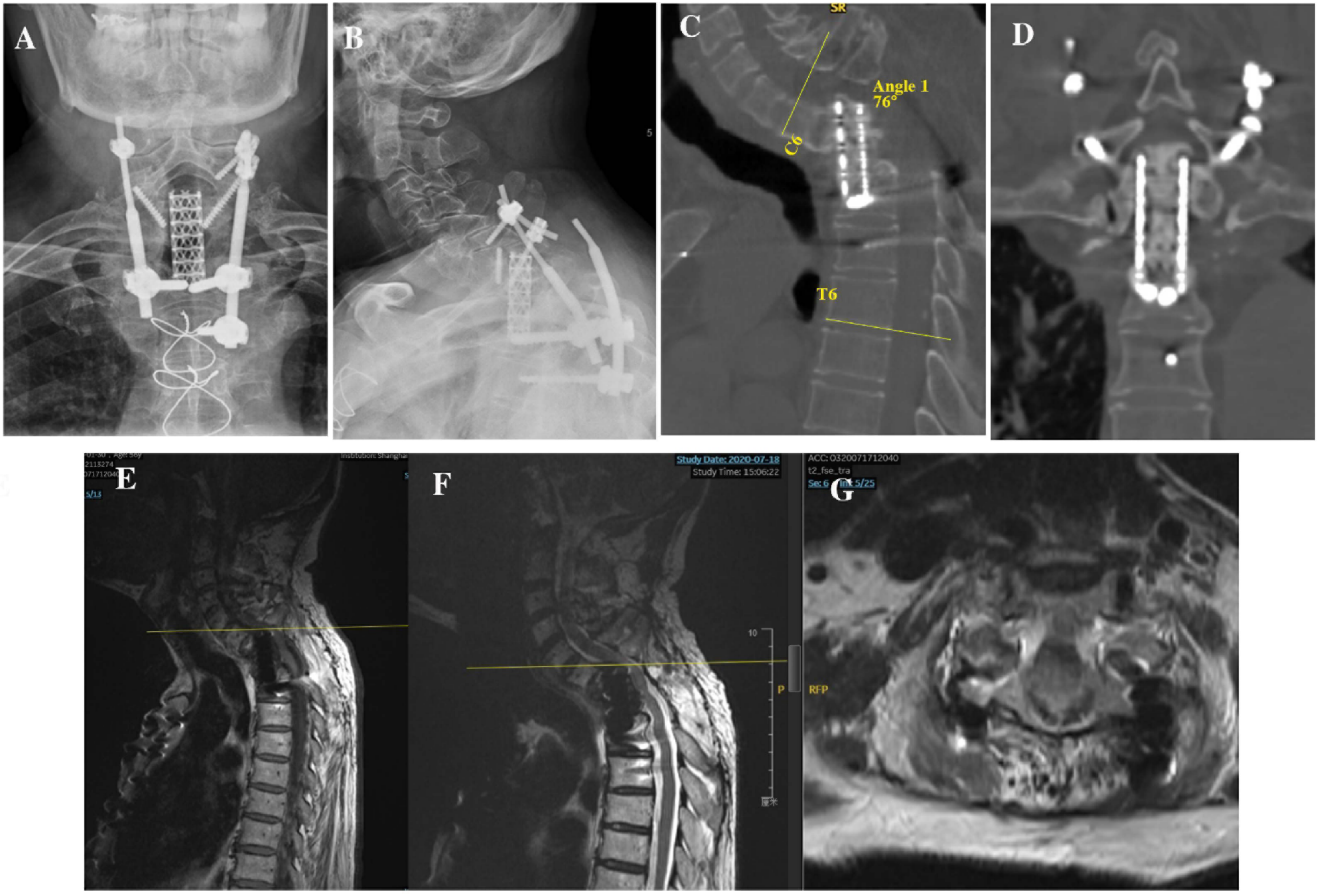
**(A,B)** Anteroposterior and lateral x-rays 5 year after first surgery showing screw/rod fracture and mesh cage displacement. **(C,D)** Sagittal and coronal CT showing increased local kyphosis and mesh cage subsidence into the C7 vertebral body. **(E–G)** T1- and T2-weighted sagittal MRIs and T2-weighted axial MRI images showed compression of spinal cord by subsided mesh cage. CT, computed tomography; MRI, magnetic resonance imaging.

In 2019, 5 years after the initial surgery, the patient experienced discomfort in the neck and shoulder following long journeys, during which he took public buses and experienced no traffic accidents. After 7 months, he experienced numbness and discomfort in both lower extremities, accompanied by fatigue, unstable walking, and abdominal girdle sensation. On admission to our hospital in 2020, an x-ray scan revealed a rod fracture and breakage of the screw in C7 (Figures 2A,B), and computed tomography (CT)/magnetic resonance imaging revealed displacement of the titanium mesh cage with its superior end almost completely subsiding into the C7 vertebral body and retropulsing into the spinal canal, thereby resulting in severe local kyphosis deformity (76° for C6-T6 cobb angel) and spinal cord compression at the C6–7 level (Figures 2C–G).

The subsided mesh cage is routinely removed to achieve direct spinal cord decompression. After discussing the surgical strategy with thoracic and vascular surgeons and considering the high risks of neurological complications and excessive bleeding to remove the titanium mesh cage, we decided to retain the mesh cage and designed a one-stage, anterior-posterior-anterior multi-approach strategy to achieve indirect decompression by correcting the kyphosis in the cervicothoracic region.

**Figure 3:**
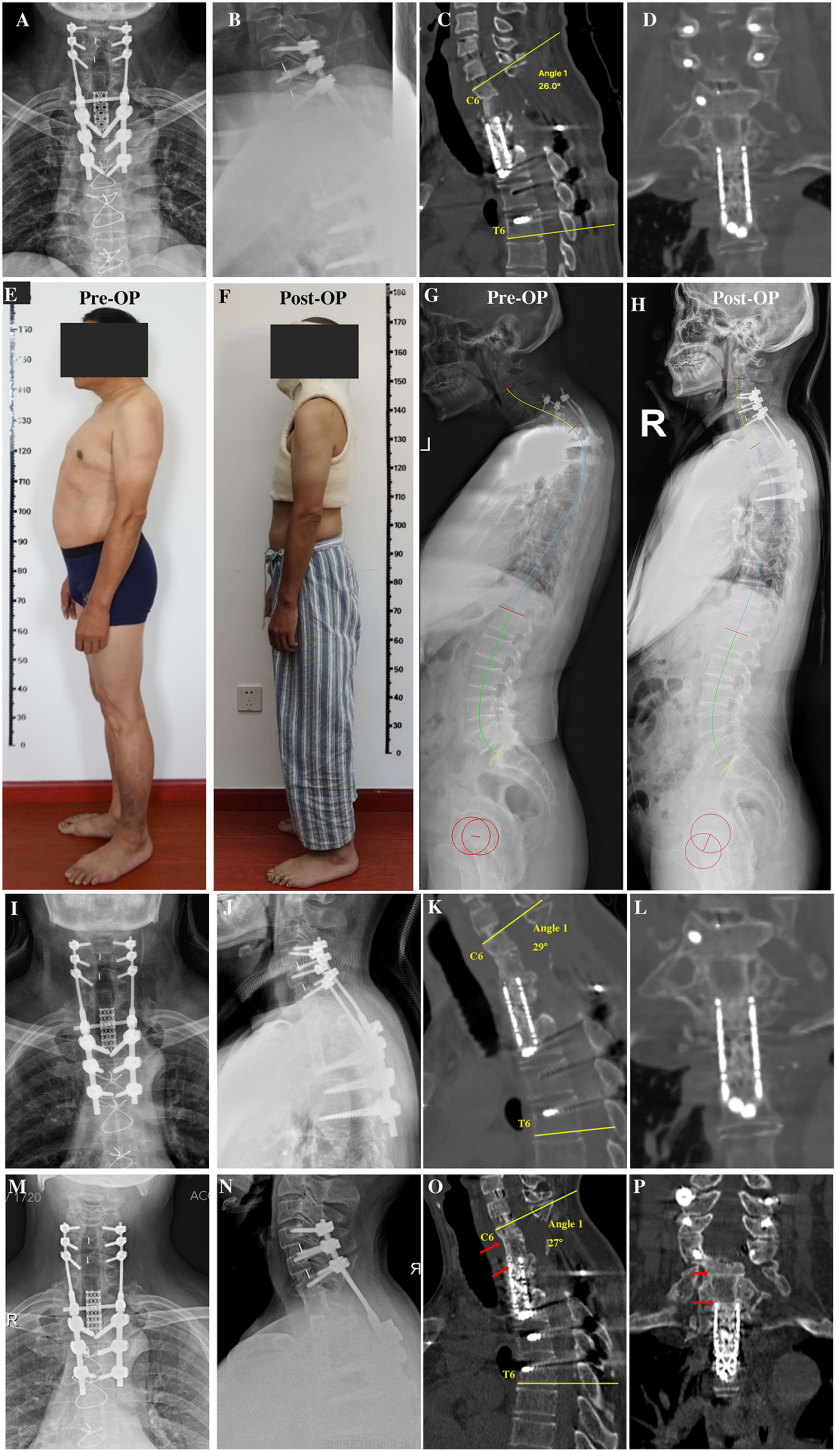
**(A,B)** Anteroposterior and lateral x-rays after revision surgery. **(C,D)** Sagittal and coronal reconstruction of CT following revision surgery showing recovery of local kyphosis. **(E)** Preoperative lateral photographs of the patient. **(F)** Lateral photograph of the patient wearing a cast postoperatively. **(G)** Preoperative full-length lateral spine radiograph of the patient. **(H)** Postoperative whole spine lateral radiograph of the patient. **(I,J)** Anteroposterior and lateral x-rays 6 months after revision surgery before removing head-neck-chest cast. **(K,L)** Sagittal and coronal CT reconstruction 6 months after revision surgery showing bone fusion between the iliac bone and mesh cage graft. **(M,N)** Anteroposterior and lateral x-rays 3 years after revision surgery showing no instrumentation failure. **(O,P)** Sagittal and coronal reconstruction of CT 3 years after revision surgery showing bone fusion among the C6 inferior endplate, iliac bone, and mesh cage graft. CT, computed tomography.

To partially recover spinal alignment and assess whether kyphosis is easy to reduce, preoperative skull traction was performed with a weight of 9 kg for 2 weeks, ultimately achieving kyphotic Cobb angle recovery of approximately 18° in the cervicothoracic region. First, we performed discectomy of C6/7 and resected the superior endplate of C7 and the rest of the vertebral body of C7 using an anterior approach to free the superior end of the mesh cage. Next, the patient was turned over, and the original instruments were removed using a posterior approach. Considering the compression of C6/7 resulting from displacement of the mesh cage, we performed decompression via bilateral laminectomy of the C6 and C7 vertebrae. Intraoperative cervical traction reduction was performed under nerve monitoring, and pedicle screws were placed at C4, C5, C6, T4, T5, and T6 to correct local kyphosis and reinforce instrumentation stability. Due to the resection of the C7 vertebra, a defect was created between the mesh head and the C6 vertebra, we turned the patient over again, and the iliac bone was harvested and placed between the inferior endplate of C6 and the superior end of the mesh cage, followed by anterior cervical discectomy and fusion for C4/5 and C5/6 to achieve long-term stability (Figure 3A–D). Postoperatively, the patient was advised to wear a head-neck-chest cast. Through these measures, the kyphosis angle at the cervicothoracic junction was corrected from 76° preoperatively to 26° postoperatively (Figure 2C). The correction of the curvature of the patient's neck can be seen on the lateral picture (Figures 3E,F). Similarly, on the lateral standing global spine x-ray, we can observe that the patient has achieved better sagittal balance (Figures 3G,H).

At 6 months postoperatively, the patient achieved symptom relief (pain diminished, numbness alleviated, and lower limb strength increased), bone fusion was observed on CT (Figures 3I–L), and the cast was then removed. At the 3-year follow-up, x-ray and CT scans revealed that bone fusion occurred among the C6 vertebra, iliac bone, and mesh cage, with no further rod/screw breakage (Figures 3M–P) and loss of kyphosis correction of whom the C6-T6 cobb angle remained 27° 3 years after surgery.

## 3 Discussion

Three-column spinal resection after total spondylectomy poses considerable challenges to the reconstruction of spinal stability. Despite the introduction of titanium-based mesh-type vertebral spacers, mechanical failure is not uncommon after total spondylectomy, with a reported risk of 27%–43% in long-term survivors (7). Radiotherapy, spine surgery, long vertebral resection, cage subsidence, and high body mass index have been confirmed as risk factors for instrumentation failure (4, 6). Failure of instrumentation includes screw loosening or backout, rod and screw fractures, and titanium mesh subsidence. The risk factors for instrumentation failure after multilevel total spondylectomy are complex. In this patient, we noticed unsatisfactory sagittal kyphotic alignment around the cervicothoracic region after the primary surgery. Uncorrected sagittal plane imbalance has been reported to predispose patients to symptomatic instrumentation failure (8). As the deformity further aggravated, abnormal kyphosis in the CTJ developed over time, thus leading to mesh cage subsidence into the C7 vertebral body and retropulsion into the spinal canal, with its superior end compressing the spinal cord. The anterior column was then shortened, which aggravated local kyphosis at the instrumented level and further enhanced the mechanical stress on the instruments, ultimately leading to rod and screw breakage. Usually, spine surgeons perform both anterior support and posterior long segmental fixation to achieve rigid stability. Long segmental fixation is comparatively conducive to the recovery of sagittal alignment, and a biomechanical finite element analysis showed that long segmental fixation can provide more rigid support than short segmental fixation; posterior reconstruction extending to at least two levels above and below the resected vertebrae has been recommended (2, 9). Thus, we concluded that one of the reasons for instrumentation failure after the first surgery in this patient was insufficient screw placement and short-segment fixation (10). Rod fractures due to local kyphosis and subsequent cage subsidence are some of the most common instrumentation failures after total spondylectomy (11). For this patient, due to inadequate understanding of kyphosis correction and spinal biomechanics at the time, as well as insufficient surgical technical skill, we used too short internal fixation devices, with flawed screw placement, and failed to fully correct local kyphotic deformity, ultimately leading to the failure of internal fixation. These issues should be thoroughly considered preoperatively.

Numerous strategies for the reconstruction of the CTJ region in degenerative spinal disease have been reported, but there is limited data on reconstruction after TES (12). Therefore, instrumentation failure of the CTJ is a significant challenge for surgeons. Previous studies have mentioned the use of multiple rods to reinforce the initial stability of the posterior instrument (13). Compared with the traditional 2-rod construct, the 4-rod technique can provide additional stability and prevent instrumentation failure. However, to the best of our knowledge, no study has reported the use of multiple rods in the cervicothoracic spine (14). Considering that fracture of the screw and rod after the first surgery may be attributed to insufficient screw placement in this patient, we decided to extend fixation to the C4, C5, C6, T5, T6, and T7 vertebrae to achieve robust posterior re-stabilisation.

A major issue to consider when mesh cage subsidence occurs is whether the cage should be removed. To date, few studies have reported the management of cage subsidence or migration after TES. Kwon et al. analysed mechanical failure after TES and reported a case of mesh cage subsidence and unilateral rod fracture. Considering the absence of spinal cord compression and the risk of additional anterior support, they eventually retained the cage and replaced the fractured rod with cobalt-chromium-based rods via a posterior approach, with further application of additional satellite rods (7). As the displaced mesh cage directly compressed the spinal cord in this patient, direct decompression by removing the mesh cage through an anterior approach may be the preferred method. However, this approach would lead to complications. For example, as the first surgery had already used the anterior approach, performing a thoracotomy was difficult because of scar adhesion. The following concern should be considered when using this method to fill a defect after removing the mesh cage: the use of a long cage does not guarantee that similar problems will not recur. Instead, previous studies have suggested using autologous free vascularised fibula grafts in the reconstruction of the mobile spine following tumour resection and have presented it as an effective reconstruction technique in the cervicothoracic spine (15). Vascularised grafts can be harvested at lengths that satisfy almost all spondylectomy defects. However, many difficulties and challenges are associated with revision surgery when vascularised grafts are used. First, owing to limited space, it is difficult to anastomose blood vessels in the cervicothoracic region. Second, one must be particularly attentive to finding the anastomotic recipient vessels. In revision surgery, the vasculature is often more difficult to dissect clearly, thus making vascular anastomosis more problematic. The posterior intercostal artery has been reported to be an appropriate donor vessel, thus allowing vascularised graft reconstruction of vertebral column defects of the lower cervical (C6–C7) and upper thoracic (T1–T3) regions (16). Thoracotomy and posterior–anterior combined surgery may be necessary to achieve anastomosis of the posterior intercostal artery and vascular leash of the graft. However, in addition to the local adhesions caused by the previous anterior surgery, the location of the aortic branch was high and close to the neck in this patient, which increased the risk of intraoperative damage to the innominate vein, thus leading to massive bleeding. Hence, these surgeries make the planning of revision surgery difficult and increase trauma in patients. Owing to these complications, we abandoned the direct anterior decompression strategy and attempted indirect decompression by restoring sagittal alignment instead of removing the mesh cage, as no subsidence had occurred at the inferior end of the mesh cage, and bone fusion was also achieved in this region. Skull traction can effectively restore spinal curvature and enhance the safety and success rates of subsequent surgeries (17). For this patient, we performed preoperative skull traction, achieved an 18° decrease in the kyphosis angle, and further decreased the kyphosis angle by 32° (58°–26° for C6-T6 cobb angle) following intraoperative traction. Notably, benefiting from the sagittal alignment reconstruction, the superior end of the titanium mesh no longer compressed the spinal cord.

Another major concern that should be considered during the strategy decision is how to fill the anterior defect between the C6 inferior endplate and the superior end of the mesh cage resulting from kyphosis correction. A shortening operation consisting of a 3-column osteotomy is necessary for cervicothoracic deformity correction (18). However, 3-column osteotomy is unsuitable for these patients, and segmental shortening without osteotomy inevitably leads to spinal cord folding and compression. To minimise trauma, the iliac bone can be used to fill the defect area. Solid bony fusion after instrumentation is one of the most crucial factors in preventing instrumentation failure (11). Fusion between the inferior end plate of C6 and the iliac bone is easy to achieve. The next issue that must be considered is how to ensure fusion between the iliac bone and superior end of the mesh cage. Before revision surgery, bone quality must be assessed. CT is commonly used to evaluate bone viability within the cage (19), and we had noticed continuous solid bone osteogenesis at the superior end of the mesh cage, which means that with the titanium mesh subsiding into the C7 vertebral body for nearly 6 years, the internal bone graft had completed creeping substitution to form bioactive new bone. Therefore, we believe that osseointegration between the C6 endplate and bone graft in the mesh cage can occur via bridging by the iliac bone. Indeed, bone reconstruction was proven using CT 3 years after revision surgery.

In conclusion, we presented a case of mesh cage subsidence and superior end retropulsion with severe spinal cord invasion in the cervicothoracic region. Our case provides an possible alternative solution that instead of removing the displaced and invalid cage, recovering the sagittal alignment to achieve indirect decompression based on unique anterior and middle column reconstruction, solid instrument construction, and bone fusion is a proven solution. Furthermore, we suggest that a similar strategy may be extended when mesh cage subsidence occurs in any region from the lower cervical spine to the lumbar spine. However, more importantly, when devising the initial surgical plan, we must fully recognize the significance of sagittal curvature, ensure a sufficiently long internal fixation, which is a crucial factor in avoiding revision surgery.

## Data Availability

The original contributions presented in the study are included in the article/Supplementary Material, further inquiries can be directed to the corresponding author.
